# Industrial LiFePO₄ battery management system dataset for early degradation analysis and remaining useful life prediction

**DOI:** 10.1016/j.dib.2026.113252

**Published:** 2026-09-11

**Authors:** MST. Asfia Binte Ulfat, Afsana Begum, Md. Abdulla Al Mamun, Sawdagar Noor Asfuq E Azam, Md. Mahedi Hassan, Imran Mahmud, A.H.M. Saifullah Sadi, Md. Shohel Sayeed

**Affiliations:** a Department of Software Engineering, Daffodil International University, Dhaka, 1216, Bangladesh; b Department of Computer Science and Engineering (CSE), Hajee Mohammad Danesh Science & Technology University, Dinajpur, Bangladesh; c GMP Lithium Ltd., Dhanmondi, Dhaka, 1209, Bangladesh; d Centre for Intelligent Cloud Computing, CoE for Advanced Cloud, Faculty of Information Science and Technology, Multimedia University, Jalan Ayer Keroh Lama, Bukit Beruang, 75450, Melaka, Malaysia

**Keywords:** Battery management system, Lithium iron phosphate battery, State of health, Remaining useful life, Battery degradation, Battery prognostics, Depth of discharge

## Abstract

This data article describes an experiment-informed R&D dataset associated with an industrial LiFePO₄ battery pack with a nominal voltage of 25.6 V and a rated capacity of 40 Ah. The dataset was prepared with industrial support from GMP Lithium Ltd., Bangladesh, and represents battery operational behaviour and capacity degradation within practical industrial operating ranges. Capacity(Ah) follows a deterministic degradation trajectory calibrated to the manufacturer’s ageing-test end points, whereas the pack-level operating variables and the eight cell voltages are structured (simulated) cycle-level values generated within the manufacturer’s specification limits; the complete generating equations are reported. The dataset consists of 3000 sequential charge-discharge cycle records in CSV format. Each record contains pack identification, cycle number, battery capacity, State of Health, Remaining Useful Life, pack voltage, pack current, a normalized lifecycle progress indicator labelled DoD(%),* operating temperature, and individual voltage measurements from eight series-connected cells. The dataset contains 17 variables, including indicators of battery degradation, pack-level operating conditions, and cell-level voltage behaviour. Supporting documentation, including a codebook, README file, and data publication permission letter, is provided with the dataset in the Mendeley Data repository. The dataset can be reused for battery degradation analysis, State of Health estimation, Remaining Useful Life prediction, assessment of cell-voltage imbalance, feature analysis, and development and evaluation of machine learning and deep learning models. The combination of capacity, operational, and cell-level voltage variables enables the study of battery ageing at both pack and cell level. It is not a raw continuous BMS log and was not sampled directly from a physical test bench; it should not be interpreted as such.

Specifications Table

**Table:** 

Subject	Engineering & Materials science
Specific subject area	Lithium Iron Phosphate battery degradation, battery health assessment, and Remaining Useful Life prediction using industrial Battery Management System data
Type of data	Table; CSV file; numerical cycle-indexed data; experiment-informed structured (simulated) and calculated data.
Data collection	The dataset is associated with a 25.6 V, 40 Ah Lithium Iron Phosphate battery pack used in an industrial environment. It was provided by GMP Lithium Ltd., Bangladesh, and was prepared using knowledge obtained from physical battery testing, Battery Management System monitoring, charge-discharge equipment, ageing tests, and battery specifications. The dataset consists of 3000 sequential charge-discharge cycle records comprising capacity, State of Health, Remaining Useful Life, pack voltage, current, a normalized lifecycle progress indicator (DoD%), temperature, and eight individual cell voltages. The dataset is a hybrid, experiment-informed resource and not a direct export from a test bench or a continuous BMS log: Capacity(Ah) follows a deterministic degradation model calibrated to GMP Lithium Ltd. ageing-test end points, and the operating and cell-voltage variables are structured (simulated) values generated within the manufacturer’s specification ranges (Sections 2.1 and 2.2.2).
Data source location	GMP Lithium Ltd., Bangladesh. The dataset is stored in the Mendeley Data repository and is affiliated with the Department of Software Engineering, Daffodil International University, Dhaka 1216, Bangladesh
Data accessibility	Repository name: Mendeley Data Data identification number: 10.17632/k675pxrd83.2 Direct URL to data: https://data.mendeley.com/datasets/k675pxrd83/2
Related research article	None

## 1 Value of the Data

• The dataset consists of 3000 consecutive operational records of an industrial 25.6 V, 40Ah LiFePO4 battery pack with Capacity(Ah), SOH(%), RUL(cycles), Voltage(V), Current(A), DoD(%)*, Temperature(C) and eight individual cell voltages.
• Data can be used for battery degradation research, State of Health estimation, Remaining Useful Life prediction, capacity fade analysis, and cell voltage imbalance within practical industrial operating ranges.
• Researchers can reuse the dataset to develop, train and compare machine learning and deep learning pipelines for battery health monitoring and predictive maintenance, with the variable-origin and collinearity information needed to avoid target leakage (Sections 4.1.3 and 4.1.8).
• The presence of pack level and cell level variables at the same time enables comprehensive analysis of the battery behavior and the variation of the individual cell voltages during the recorded lifecycle.
• Use of the dataset for feature selection, correlation analysis, anomaly detection, statistical analysis and time-series visualization in battery management and energy storage research.
• The data are provided in CSV format, with a codebook and README file, making the data easy to reuse in Python, R, MATLAB and other data analysis environments.

## 2 Background

Lithium Iron Phosphate (LFP) batteries are extensively used in electric vehicles, renewable energy storage systems, backup power applications and industrial energy systems due to their long cycle life, thermal stability and operational safety [1,2]. Despite these advantages, repeated charge/discharge gradually decreases battery capacity and affects the overall performance. The monitoring of this degradation is needed for the estimation of State of Health and prediction of Remaining Useful Life in Battery Management Systems [[3], [4], [5]].

Most of the available battery datasets are generated under controlled laboratory conditions. Such datasets are useful for model development but may not fully represent the variation in practical industrial operation. Battery degradation, State of Health estimation and Remaining Useful Life prediction have been studied using existing datasets for different battery chemistries and operating profiles [[6], [7], [8]]. Data-driven approaches that estimate SoH and RUL from readily available voltage-based features, for example support vector regression trained on first-life data and on-board voltage measurements [9], depend on the availability of well-documented, cycle-indexed datasets in which the origin of every variable is explicit. Actual data from the operation can include voltage, current, physical depth of discharge, temperature and single cell behaviour changes over the lifetime of battery.

This dataset was prepared to provide structured industrial Battery Management System records associated with a 25.6 V, 40 Ah LiFePO4 battery pack. The data were provided by GMP Lithium Ltd., Bangladesh, and include 3000 sequential charge-discharge cycle records. The dataset includes pack-level measurements and individual cell voltages, and was prepared using knowledge obtained from physical battery testing, BMS monitoring, charge-discharge equipment, ageing tests, and battery specifications. It was structured and documented to enable battery degradation analysis, State of Health estimation, Remaining Useful Life prediction, cell-level assessment, and machine learning research using operational data bounded by the manufacturer’s specification [10]. The pack-level voltage boundaries used to structure the dataset (nominal 25.6 V; charge range 28.0–29.2 V; recommended operating range 23.2–29.2 V) are taken from the manufacturer’s specification sheet; the alignment of a battery bank’s nominal and fully-charged voltages is a system-level parameter that affects energy-transfer efficiency and battery lifespan in PV–battery installations [11], which is why these boundaries are documented explicitly in Section 2.2.2.

## 3 Data Description

### 3.1 Description

This dataset is provided as a comma separated value file and is named ‘GMP_LFP_40Ah_3000Cycle.csv’. The dataset consists of 3000 sequential cycle records from one industrial LiFePO₄ battery pack with a nominal voltage of 25.6 V and capacity of 40 Ah. Each row represents one battery cycle, and the columns are battery degradation, pack-level operating conditions, and cell voltages [10].

Cycle records are ordered from Cycle 1 to Cycle 3000. During the recorded lifecycle the battery capacity drops from about 40 Ah to 28 Ah. State of Health ranges from approximately 100% to 70%. Remaining Useful Life decreases from 2999 cycles to zero. Pack-level variables consist of: Voltage, Current, the normalized lifecycle progress indicator DoD(%)*, and operating temperature.The cell level measurements are the voltage of eight individual cells. These are recorded in Cell1_V to Cell8_V.

The repository contains a codebook with the variables and their units, a README file that explains the structure of the data set and its reuse, and a permission letter that allows the publication of the industrial data.

*Throughout this article, DoD(%) denotes a normalized lifecycle progress indicator, calculated as (CycleNumber / 3000) × 100, and **not** the physical depth of discharge of an individual charge–discharge cycle. The column name is retained for compatibility with the Mendeley Data record (see Section 4.1.6).

The Table 1(b) above clarifies the origin of each column. Voltage(V), Current(A), Temperature(C), and Cell1_V–Cell8_V are structured (simulated) cycle-level values generated within the specification ranges supplied by GMP Lithium Ltd. and calibrated to the operating behaviour observed on the BMS; the generating equations are given in Section 2.2.2. Capacity(Ah) follows a deterministic power-law degradation trajectory (Eq. (1)) whose end points (40 Ah at Cycle 1; 28 Ah, i.e. 70% SOH, at Cycle 3000) and curvature were calibrated to BMS capacity readings and to GMP Lithium Ltd.’s ageing-test data. SOH (%), RUL (cycles), and DoD (%) are calculated variables derived from Capacity or CycleNumber and are perfectly collinear with their source variables. Pack-level values (Voltage, Current, Temperature) are cycle-level representative values; they are not synchronized measurements captured at a single timestamp within the cycle, and no snapshot instant was selected (Section 2.2.2). Cell voltages were generated from the same statistical distribution for all eight cells and do not carry independent cell-level ageing signals; cell-level analysis is limited to voltage imbalance (the spread across the eight cells per cycle). Because the operating and cell-voltage variables were generated independently of CycleNumber, they carry no information about capacity fade by construction; this is quantified in Table 5 and must be considered when selecting predictive features (Section 2.2.7).

**Table 1b:** Variable origin and interpretation: measured, calculated, and structured columns in the dataset.

Variable	Origin	Sampling / Generation Basis	Notes on interpretation
PackID	Structured	Constant value (1) assigned to all records	Dataset identifier; carries no measurement information
CycleNumber	Structured	Integer sequence 1–3000	Ordered lifecycle index; not a BMS timestamp
Capacity(Ah)	BMS-informed / R&D	Deterministic power-law fade trajectory, Capacity = 40 − 12·(CycleNumber/3000)1.35 (Eq. (1)), calibrated to BMS capacity readings and ageing-test end points (40 Ah at Cycle 1; 28 Ah at Cycle 3000)	Decreases monotonically from 40 Ah to 28 Ah across the lifecycle; loss per cycle increases with cycle number
SOH(%)	Calculated	SOH = [Capacity(Ah) / 40 Ah] × 100	Direct transformation of Capacity; perfectly collinear with Capacity(Ah)
RUL(cycles)	Calculated	RUL = 3000 − CycleNumber	Retrospective countdown label; perfectly collinear with CycleNumber. Not a probabilistic end-of-life estimate.
Voltage(V)	BMS-informed / structured (simulated)	Structured (simulated) cycle-level value drawn from N(25.60 V, 0.12 V) (Eq. (5)), calibrated to the pack voltage observed on the BMS during operation and lying within the recommended operating range (23.2–29.2 V); one representative value per cycle, not a waveform or a timestamped snapshot	Stationary across the lifecycle; no dependence on CycleNumber (Table 3, Table 5)
Current(A)	BMS-informed / structured (simulated)	Structured (simulated) cycle-level value drawn from a uniform distribution U(−40 A, +40 A) (Eq. (6)) spanning the specified charging (10–20 A) and discharging (20–40 A) currents and the 40 A BMS continuous limit; positive = charge, negative = discharge	Mean ≈ 0 A (−0.42 A) because charge and discharge values are equally likely under Eq. (6)
DoD(%)	Calculated	(CycleNumber / 3000) × 100	Normalized lifecycle progress indicator*. NOT physical depth of discharge of an individual cycle. Column name retained for repository compatibility.
Temperature(C)	BMS-informed / structured (simulated)	Structured (simulated) cycle-level value drawn from N(28.0 °C, 1.8 °C) (Eq. (7)), calibrated to the pack temperature observed from the BMS MOS and battery sensors; all values within 21–34 °C and within the specified 0–60 °C charging range	No systematic trend with degradation observed across lifecycle.
Cell1_V – Cell8_V	BMS-informed / structured (simulated)	Independent draws from N(3.200 V, 0.008 V) for each cell and each cycle (Eq. (8)); no cell-specific ageing term	Supports cell-voltage imbalance (spread) analysis only; carries no individual cell degradation trajectory (Section 2.2.2)

### 3.2 Repository file structure

GMP_LFP_40Ah_3000Cycle.csv

Main dataset containing the 3000 battery-cycle records.

DataSet_CodeBook.pdf

Description of the variable names, units, data types, and meanings.

README.docx

General description of the dataset, file structure, and reuse instructions.

Data_Publication_Permission_Letter_GMP.pdf

Permission from GMP Lithium Ltd. authorizing academic publication and sharing of the dataset.

GMP_Specs_LFP_25.6V_40AH.pdf

GMP Industrial LFP Battery Original Manufacturer Specification Sheet for the GMP-LFP-2500 pack (25.6 V, 40 Ah, 8S LiFePO₄), provided by GMP Lithium Ltd. It documents the nominal, charge and operating voltage ranges, individual cell voltage limits, standard charge/discharge currents, BMS continuous and peak current limits, balancing current and operating temperature range that define the specification boundaries used to structure the dataset (Section 2.2.2). Table 2 presents the minimum, maximum, mean, and standard deviation of the numerical variables in the dataset. The statistics summarize the recorded ranges of the degradation indicators, pack-level operating variables, and individual cell voltages across the 3000 battery cycles. The constant PackID variable was excluded from the table because all records belong to the same battery pack.

**Table 2:** Descriptive statistics for all numerical variables (n = 3000 cycles).

Variable	Minimum	Maximum	Mean	Standard deviation
CycleNumber	1.00	3000.00	1500.50	866.17
Capacity(Ah)	28.00	40.00	34.89	3.58
SOH(%)	70.00	100.00	87.23	8.96
RUL(cycles)	0.00	2999.00	1499.50	866.17
Voltage(V)	25.21	26.07	25.60	0.12
Current(A)	−40.00	39.96	−0.42	23.05
DoD(%)	0.03	100.00	50.02	28.87
Temperature(C)	21.09	34.35	27.97	1.87
Cell1_V	3.173	3.229	3.200	0.008
Cell2_V	3.173	3.224	3.200	0.008
Cell3_V	3.171	3.229	3.200	0.008
Cell4_V	3.174	3.226	3.200	0.008
Cell5_V	3.172	3.230	3.200	0.008
Cell6_V	3.174	3.227	3.200	0.008
Cell7_V	3.169	3.227	3.200	0.008
Cell8_V	3.172	3.236	3.200	0.008

Table 3 summarizes the mean, standard deviation (SD), skewness and interquartile range (IQR) of the principal variables within the three lifecycle phases defined in Fig. 2 (early life, Cycles 1–1000; mid life, Cycles 1001–2000; end of life, Cycles 2001–3000). Capacity(Ah) and SOH(%) shift downward from phase to phase and their within-phase spread increases towards end of life because the degradation trajectory steepens with cycle number (Eq. (1)). In contrast, the pack-level operating variables and the cell-voltage imbalance have essentially the same mean, dispersion and shape in all three phases, i.e. they are stationary over the recorded lifecycle.

**Table 3:** Descriptive statistics of the principal variables by lifecycle phase (n = 1000 cycles per phase). Skewness is the bias-corrected sample skewness; IQR = Q3 − Q1.

Variable	Lifecycle phase (cycles)	Mean	SD	Skewness	IQR
Capacity(Ah)	Early life (1–1000)	38.84	0.81	−0.267	1.43
Capacity(Ah)	Mid life (1001–2000)	35.25	1.22	−0.082	2.11
Capacity(Ah)	End of life (2001–3000)	30.59	1.46	−0.049	2.53
SOH(%)	Early life (1–1000)	97.10	2.04	−0.267	3.57
SOH(%)	Mid life (1001–2000)	88.12	3.05	−0.082	5.29
SOH(%)	End of life (2001–3000)	76.47	3.66	−0.049	6.32
Voltage(V)	Early life (1–1000)	25.602	0.118	0.117	0.155
Voltage(V)	Mid life (1001–2000)	25.609	0.120	−0.049	0.160
Voltage(V)	End of life (2001–3000)	25.601	0.118	0.061	0.157
Current(A)	Early life (1–1000)	−0.77	22.90	0.041	38.84
Current(A)	Mid life (1001–2000)	−0.09	23.18	0.046	39.77
Current(A)	End of life (2001–3000)	−0.39	23.08	0.035	40.61
Temperature(C)	Early life (1–1000)	27.94	1.83	0.074	2.45
Temperature(C)	Mid life (1001–2000)	27.94	1.90	0.025	2.63
Temperature(C)	End of life (2001–3000)	28.02	1.89	−0.015	2.54
Cell-voltage imbalance (V)	Early life (1–1000)	0.0228	0.0065	0.341	0.0086
Cell-voltage imbalance (V)	Mid life (1001–2000)	0.0227	0.0063	0.348	0.0086

Table 4 lists the individual summary statistics of the eight cell-voltage columns. The eight means agree to within 0.4 mV, the standard deviations range from 0.0077 V to 0.0082 V, medians coincide with the means, and skewness lies within ±0.07 for every cell. A one-way ANOVA (F = 0.71, p = 0.665) and a Kruskal–Wallis test (H = 5.22, p = 0.633) across the eight columns do not indicate any difference in central tendency; Levene’s test indicates a small but statistically detectable difference in dispersion (p = 0.008), consistent with the narrow range of standard deviations. These statistics quantify the cell uniformity that is visible in Fig. 5(a); the uniformity follows from the generation model of Section 2.2.2, in which all eight cells share the same distribution and no cell-specific ageing term is present.

**Table 4:** Individual summary statistics for each cell-voltage column, Cell1_V to Cell8_V (n = 3000 cycles each).

Cell	Mean (V)	SD (V)	Min (V)	Max (V)	Median (V)	Skewness
Cell1_V	3.2002	0.0080	3.1730	3.2289	3.2003	−0.045
Cell2_V	3.1999	0.0077	3.1734	3.2240	3.2000	−0.006
Cell3_V	3.2000	0.0080	3.1708	3.2288	3.1998	−0.041
Cell4_V	3.1999	0.0078	3.1741	3.2257	3.1999	0.023
Cell5_V	3.2000	0.0080	3.1717	3.2295	3.2002	−0.055
Cell6_V	3.2000	0.0079	3.1744	3.2274	3.2000	0.012
Cell7_V	3.1999	0.0082	3.1686	3.2270	3.2000	0.010
Cell8_V	3.2001	0.0079	3.1720	3.2358	3.2001	0.065

Table 5 complements the Pearson heatmap (Fig. 4) with Spearman rank correlation coefficients (ρ) and mutual information (MI) estimates between each operating variable and Capacity(Ah), and equivalently CycleNumber. Spearman ρ detects any monotonic relationship and MI any statistical dependence, linear or non-linear. For Voltage(V), Current(A), Temperature(C), the cell-voltage imbalance and every individual cell voltage, |ρ| ≤ 0.03 (all p > 0.1) and MI ≈ 0 nats, whereas CycleNumber has ρ = −1.000 and MI = 6.415 nats with Capacity(Ah). The non-linear analysis therefore leads to the same conclusion as the linear analysis: in this dataset the operating conditions are statistically independent of capacity fade, as expected from the generation model (Section 2.2.2).

**Table 5:** Spearman rank correlation (ρ, with two-sided p-value) and mutual information (MI, nats; k-nearest-neighbour estimator, k = 3) between operating variables and Capacity(Ah) and CycleNumber (n = 3000). MI values below 0.001 are shown as 0.000.

Variable	ρ vs Capacity(Ah)	p-value	MI vs Capacity(Ah)	ρ vs CycleNumber	MI vs CycleNumber
CycleNumber	−1.000	< 0.001	6.415	+1.000	6.571
Voltage(V)	−0.000	0.996	0.011	+0.000	0.002
Current(A)	−0.007	0.692	0.028	+0.007	0.024
Temperature(C)	−0.028	0.121	0.000	+0.028	0.000
Cell-voltage imbalance (V)	+0.009	0.606	0.000	−0.009	0.000
Cell1_V	+0.022	0.228	0.007	−0.022	0.019
Cell2_V	+0.019	0.291	0.013	−0.019	0.013
Cell3_V	+0.007	0.715	0.000	−0.007	0.000
Cell4_V	−0.010	0.586	0.000	+0.010	0.000
Cell5_V	+0.015	0.399	0.000	−0.015	0.000
Cell6_V	+0.019	0.294	0.000	−0.019	0.000
Cell7_V	−0.003	0.865	0.000	+0.003	0.000
Cell8_V	+0.012	0.513	0.000	−0.012	0.000

Table 6 reports formal normality tests for the six distributions shown in Fig. 3. Pack Voltage(V) and Temperature(C) are consistent with a normal distribution under all three tests (Shapiro–Wilk, Kolmogorov–Smirnov and D’Agostino–Pearson K²; all p > 0.2). Capacity(Ah), SOH(%) and Current(A) are non-normal and platykurtic (excess kurtosis ≈ −1.2), as expected for a monotonic trajectory and for a uniform-type distribution, respectively. The cell-voltage imbalance is mildly right-skewed (skewness = 0.35), the expected shape of the range of eight independent normal values; the Shapiro–Wilk and K² tests reject normality (p < 0.001) while the KS test does not (p = 0.127), which reflects the higher power of the former tests at n = 3000.

**Table 6:** Normality tests for the distributions shown in Fig. 3 (n = 3000). SW = Shapiro–Wilk; KS = Kolmogorov–Smirnov against a normal distribution with mean and SD estimated from the sample; K² = D’Agostino–Pearson omnibus test. Skewness and excess kurtosis are bias-corrected sample values. Verdict at α = 0.05.

Variable	SW W	SW p	KS D	KS p	K²	K² p	Skewness	Excess kurtosis	Normality
Capacity(Ah)	0.9389	< 0.001	0.0805	< 0.001	2162.33	< 0.001	−0.267	−1.181	Rejected
SOH(%)	0.9389	< 0.001	0.0805	< 0.001	2162.33	< 0.001	−0.267	−1.181	Rejected
Voltage(V)	0.9995	0.655	0.0096	0.940	1.93	0.382	0.042	0.090	Not rejected
Current(A)	0.9549	< 0.001	0.0582	< 0.001	2329.11	< 0.001	0.041	−1.192	Rejected
Temperature(C)	0.9995	0.599	0.0189	0.230	0.69	0.709	0.027	0.047	Not rejected
Cell-voltage imbalance (V)	0.9927	< 0.001	0.0214	0.127	59.28	< 0.001	0.346	0.143	Rejected (SW, K²)

Table 7 quantifies the outliers indicated by the boxplots in Fig. 5(a) using the Tukey rule, i.e. values below Q1 − 1.5·IQR or above Q3 + 1.5·IQR. Between 15 and 24 values per cell (0.50–0.80%) fall outside the fences, 157 values (0.65%) in total across the 24,000 cell-voltage entries, and 23 cycles (0.77%) have a cell-voltage imbalance above the upper fence of 0.040 V (none below the lower fence). All flagged values lie well inside the specified individual cell voltage range (2.50–3.65 V) and were retained (Section 4.2.4).

**Table 7:** Number and percentage of outliers per variable according to the Tukey 1.5·IQR rule (fences computed from n = 3000 values per column).

Variable	Lower fence	Upper fence	Below	Above	Total	Percentage
Cell1_V	3.1789	3.2214	15	9	24	0.80%
Cell2_V	3.1788	3.2211	7	8	15	0.50%
Cell3_V	3.1776	3.2224	14	6	20	0.67%
Cell4_V	3.1784	3.2209	11	12	23	0.77%
Cell5_V	3.1776	3.2223	12	6	18	0.60%
Cell6_V	3.1787	3.2212	11	8	19	0.63%
Cell7_V	3.1774	3.2223	9	10	19	0.63%
Cell8_V	3.1791	3.2214	8	11	19	0.63%
Cell-voltage imbalance (V)	0.0050	0.0400	0	23	23	0.77%
All cell voltages (24,000 values)	—	—	87	70	157	0.65%

## 4 Experimental Design, Materials and Methods

To state the origin of the data unambiguously: this dataset was not sampled directly from a physical test bench, and it is not a continuous export from the Battery Management System (BMS). It is a hybrid, experiment-informed R&D dataset, and two categories of columns must be distinguished. (i) CycleNumber, Capacity(Ah), SOH(%), RUL(cycles) and DoD(%)* are deterministic: Capacity(Ah) follows a single power-law degradation trajectory whose end points (40 Ah at Cycle 1 and 28 Ah, i.e. 70% SOH, at Cycle 3000) and curvature were calibrated by GMP Lithium Ltd. to BMS capacity readings and accelerated ageing-test results for the 25.6 V, 40 Ah pack, and SOH(%), RUL(cycles) and DoD(%) are exact transformations of Capacity(Ah) or CycleNumber. (ii) Voltage(V), Current(A), Temperature(C) and Cell1_V–Cell8_V are structured (simulated) cycle-level values generated as independent pseudo-random draws from distributions whose parameters were set from the manufacturer’s specification sheet (GMP_Specs_LFP_25.6V_40AH.pdf) and from the operating behaviour observed on the JK BMS; they are not individual BMS measurements. The exact equations, distribution parameters and pseudo-code are given in Section 2.2.2, and a variable-by-variable classification is given in Table 1(b). Throughout this article the terms “structured” and “simulated” are used with this meaning.

### 4.1 Battery pack and data source

The dataset was prepared with industrial support from GMP Lithium Ltd., Bangladesh. The company permitted the dataset to be used for academic research, journal publication, and public sharing through a recognized research data repository. The dataset is an experiment-informed R&D resource: Capacity(Ah) follows a deterministic degradation trajectory calibrated to ageing-test data, and the pack-level and cell-level operating variables were structured (simulated) within the specification ranges of GMP Lithium Ltd.’s industrial 25.6 V, 40 Ah LiFePO₄ battery pack and calibrated to the operating behaviour observed on the BMS, so that the complete 3000-cycle lifecycle is represented.A variable-by-variable classification of measured, calculated, and structured columns is provided in Table 1(b).

The dataset was prepared by GMP Lithium Ltd.’s internal R&D team using their physical battery testing equipment, BMS monitoring systems, charge-discharge apparatus, and battery specification records. The authors of this article are responsible for structuring, documenting, and publishing the dataset as an academic resource. The CRediT “Data Curation” role attributed to the first author reflects the academic structuring, cleaning, and documentation of the data provided by GMP Lithium Ltd., not the original data generation. GMP Lithium Ltd. has granted written permission for academic publication and confirms responsibility for the accuracy of the underlying battery specification and operational records.

This dataset is an experiment-informed R&D resource prepared using knowledge obtained from physical battery testing, BMS monitoring, charge-discharge equipment, accelerated ageing tests, practical operating experience, and battery specifications. It is not a direct timestamp-by-timestamp export from a single continuous BMS logging session. It is not a direct timestamp-by-timestamp export from a single continuous BMS logging session, nor a set of values sampled directly from a test bench. The dataset comprises a total of 3000 sequential charge-discharge cycle records. Each record contains battery degradation indicators, pack-level operating variables, and individual cell-voltage measurements [10]. The dataset was prepared for battery degradation analysis, State of Health assessment, Remaining Useful Life prediction, and cell-level voltage evaluation.

#### 4.1.1 Data acquisition setup

A JK Battery Management System from JIKONG was used to monitor the battery pack. The BMS model was JK_BD4A20S6P, supporting battery configuration from 8 to 20 cells in series. For the present battery pack, the system was configured with eight LiFePO₄ cells in series.

The BMS employs active cell balancing with a rated balancing current of 0.4 A. The manufacturer’s label specifies it supports 60 A continuous, 100 A peak current. The BMS was employed to track pack-level operating conditions and individual cell voltages during repeated charge/discharge operation. The supported current range and balancing current parameters of the BMS model in this study are directly obtained from the original manufacturer’s product label, and no independent third-party calibration test has been carried out. The 60 A continuous and 100 A peak values are the hardware ratings printed on the BMS module label; the pack-level specification sheet (GMP_Specs_LFP_25.6V_40AH.pdf) lists the BMS current limits configured for the GMP-LFP-2500 pack as 40 A continuous and 80 A peak, and the ±40 A range of the Current(A) column corresponds to the configured continuous limit.

The pack-level operating variables are Voltage (V), Current (A) and Temperature (C). Cell1_V to Cell8_V are the individual cell-voltage variables; all of these are structured values (Section 2.2.2). Battery degradation and lifecycle variables were represented by Capacity (Ah), SOH (%) and RUL (cycles).

The collected records were sorted in the order of CycleNumber from 1 to 3000 and saved in CSV format. The BMS model, supported cell range, balancing current, continuous current and peak current stated above are taken from the BMS manufacturer’s label (see the note at the end of the previous paragraph).

#### 4.1.2 Recorded measurements and variable calculation

Each record in the dataset represents one battery cycle. The variables were grouped into groups of lifecycle and degradation variables, pack level operating variables and individual cell-voltage variables.

#### 4.1.3 Generation of the operating variables: equations and pseudo-code

The pack-level operating variables Voltage(V), Current(A) and Temperature(C) and the eight cell-voltage columns Cell1_V to Cell8_V are the operating variables of the R&D dataset. Their permissible ranges and typical operating levels were established from the JK BMS monitoring of the 25.6 V, 40 Ah pack (pack voltage, pack current, MOS and battery temperature sensors and individual cell voltages are all reported by the BMS) and from the manufacturer’s specification sheet; the column values themselves were then structured (simulated) as independent cycle-level draws from the calibrated distributions given in Section 2.2.2. Each row therefore holds one representative value per variable per cycle. Because these are cycle-level representative values rather than samples of a measured waveform, no snapshot instant (end-of-charge, mid-discharge, random or otherwise) was selected: the values within a row are not synchronized measurements at a common timestamp, and they must not be interpreted as instantaneous BMS readings at a defined point within the charge–discharge profile. Consequently, Voltage(V) and Current(A) in the same row do not represent a simultaneous operating point, and the Current(A) sign only indicates the representative direction (positive = charge, negative = discharge) assigned to that cycle.Capacity(Ah) represents the available battery capacity associated with each cycle. CycleNumber identifies the sequential position of the record, ranging from 1 to 3000. PackID identifies the battery pack and remains constant because all records belong to the same pack.

**Mathematical synthesis logic:**

To satisfy the reproducibility and reusability requirement of Data in Brief, the complete mathematical synthesis logic used to generate the dataset is given here, followed by the pseudo-code and the random seed. Let n = CycleNumber ∈ {1, 2, …, 3000}, N = 3000 and C_0_ = 40 Ah (rated capacity).

**Deterministic columns:**

(1) $$\text{Capacity}\left(\text{Ah}\right)n=C_{0}-\Delta C\cdot {\left(n/N\right)}^{k},\text{with}\Delta C=12\text{Ah}\;\text{and}\;k=1.35$$

(2) $$\text{SOH}\left(\%\right)n=\text{Capacity}\left(\text{Ah}\right)n/C_{0}\times 100$$

(3) RUL(cycles)n=N−n

(4) DoD(%)n=n/N×100

Eq. (1) is a single-term power-law fade model. Its three constants were fixed by GMP Lithium Ltd. from the ageing-test end points of the pack (100% SOH at beginning of life; 70% SOH, i.e. 28 Ah, after 3000 cycles) and from the observation that the capacity loss per cycle increased over the life of the pack; the exponent k = 1.35 > 1 reproduces this accelerating loss (≈ 0.24 mAh in the first cycle and ≈ 5.4 mAh in the last). Eq. (1) reproduces the published Capacity(Ah) column to within 1 × 10−8 Ah, and Eqs. (2)–(4) reproduce SOH(%), RUL(cycles) and DoD(%) exactly. Under Eq. (1), SOH crosses the 80% level between Cycle 2221 and Cycle 2222 (Fig. 1).

**Fig. 1:**
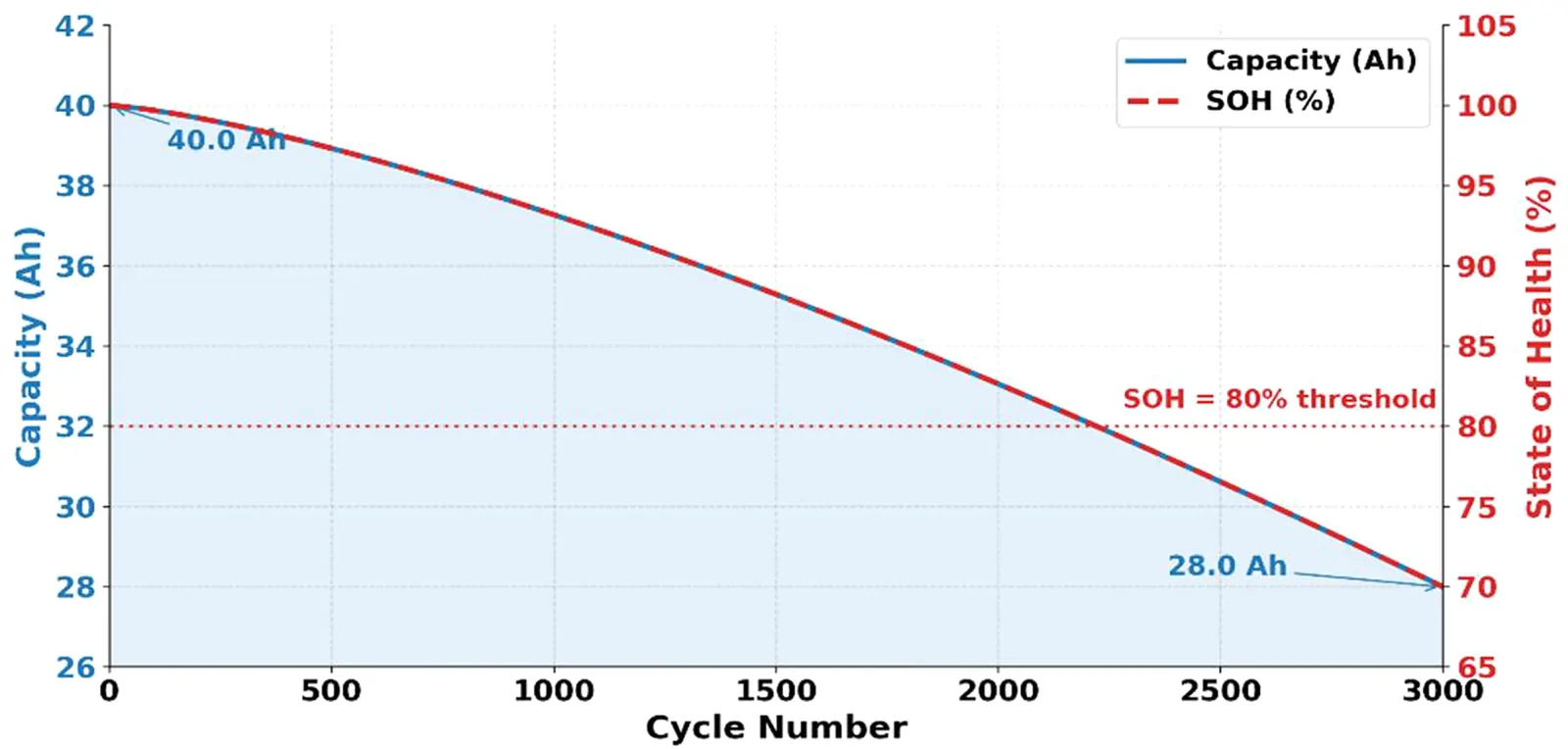
Capacity and State of Health (SOH) across 3000 battery, charge-discharge cycles.

Structured (stochastic) columns. For each cycle n, the operating variables were generated as mutually independent pseudo-random draws, independent of n and of one another:

(5) Voltage(V)n∼N(μV=25.60V,σV=0.12V)

(6) Current(A)n∼U(−40A,+40A)

(7) $$\text{Temperature}\left(C\right)n∼N\left(\mu T=28.0^{\circ }C,\sigma T=1.8^{\circ }C\right)$$

(8) Celli_Vn∼N(μc=3.200V,σc=0.008V),i=1,…,8,independentpercell

where N(μ, σ) denotes a normal distribution with mean μ and standard deviation σ, and U(a, b) a continuous uniform distribution on [a, b]. The parameters were chosen from the specification sheet and from the BMS observations as follows. μV is the nominal pack voltage (8 × 3.2 V); σV was set so that ±3σ (25.24–25.96 V) spans the pack-voltage plateau observed on the BMS during normal operation, well inside the recommended operating range of 23.2–29.2 V. The current range ±40 A corresponds to the 40 A BMS continuous current limit configured for the pack and covers the standard charging (10–20 A) and discharging (20–40 A) currents; a uniform distribution was used because no single operating current dominated the recorded operation, and the sign convention is positive = charge, negative = discharge. μT and σT reflect the pack temperature observed in the indoor operating environment (approximately 21.7–34.3 °C over ±3.5σ), within the specified 0–60 °C charging range. μc is the nominal cell voltage and σc reproduces the cell-to-cell scatter observed on the BMS cell-voltage display (typically within ±20 mV) under active balancing at 0.4 A. No generated value fell outside the specification limits, so no clipping or rejection was applied, and the stochastic columns are therefore stationary, mutually independent and independent of the degradation trajectory by construction. The CSV file stores the values with up to eight decimal places.

**Pseudo-code:**

**Table:** 

import numpy as np, pandas as pd
np.random.seed(42) # legacy NumPy RandomState (MT19937)
N = 3000; n = np.arange(1, N + 1)
a = 12.0 / N ** 1.35
Capacity = 40.0 − a · n ** 1.35 # Eq. (1)
SOH = Capacity / 40.0 · 100 # Eq. (2)
RUL = N − n # Eq. (3)
DoD = n / N · 100 # Eq. (4)
Voltage = np.random.normal(25.60, 0.12, N) # Eq. (5) 1st draw
Current = np.random.uniform(−40.0, 40.0, N) # Eq. (6) 2nd draw
Temperature = np.random.normal(28.0, 1.8, N) # Eq. (7) 3rd draw
Cells = np.random.normal(3.200, 0.008, (N, 8)) # Eq. (8) 4th draw; col i → Cell{i}_V
PackID = 1
assemble 17 columns in the order of Table 1(a); write GMP_LFP_40Ah_3000Cycle.csv

The generation run was carried out by GMP Lithium Ltd.’s R&D team in Python with NumPy and pandas, using the legacy NumPy global random number generator (Mersenne Twister MT19937) initialised with np.random.seed(42). The four stochastic draws are made in the order Voltage → Current → Temperature → cell-voltage block, exactly as listed in the pseudo-code; because the generator state is shared, this order is part of the specification. Re-running the script reproduces every value of the published CSV file: the maximum absolute difference between the regenerated and published columns is 5 × 10−9, which is the rounding of the CSV file itself. The complete script is provided with this article as Supplementary File 1 (GMP_LFP_generation_model.py, with an identical Jupyter notebook), and it also verifies Eqs. (1)–(4) directly against the published file.

Cell-level uniformity and its consequences for model training. Eq. (8) assigns the same distribution to all eight cells and contains no cell-specific ageing term, so the cell-voltage columns are stationary over the 3000 cycles (Table 3), uniform across cells (Table 4) and uninformative about degradation (Table 5). Real LiFePO₄ packs exhibit non-uniform cell ageing, local thermal gradients and capacity divergence; these effects were deliberately not injected because GMP Lithium Ltd. did not hold cell-by-cell capacity or impedance measurements for this pack (the JK BMS reports cell voltages and their difference, not individual cell capacities), and inventing cell-specific degradation trajectories without a measurement basis would have produced signals that look physical but are unsupported by data. The consequences for machine-learning use are as follows: (i) models that use Cell1_V–Cell8_V or the cell-voltage imbalance as inputs will learn no relationship with SOH, RUL or Capacity from this dataset, and any apparent contribution of these features indicates over-fitting to noise; (ii) the columns are suitable for developing and testing cell-imbalance detection logic, data-quality checks and alarm thresholds (for example imbalance > 40 mV, the upper Tukey fence in Table 7), for feature-engineering pipelines, and as a null-signal baseline against which real cell-divergence data can be compared; (iii) the dataset must not be used to train or benchmark models of cell-to-cell divergence or of individual cell degradation trajectories. Users who require such signals should combine this dataset with a cell-level ageing dataset such as those in [[6], [7], [8]].

#### 4.1.4 State of health (SOH) calculation

State of Health was calculated by comparing the available capacity at each cycle with the rated battery capacity of 40 Ah:

SOH(%)=[Capacity(Ah)/40Ah]×100

For example, a measured capacity of 36 Ah corresponds to an SOH value of 90%.

#### 4.1.5 Remaining useful life (RUL) calculation

Remaining Useful Life was defined as the number of cycles remaining to the last recorded cycle:

RUL(cycles)=3000−CycleNumber.

For example, the value of the corresponding RUL (cycles) is 2000 at CycleNumber 1000. At the final cycle, CycleNumber 3000, the RUL value is 0.

#### 4.1.6 Depth of discharge (DoD) calculation

The column labelled DoD (%)* in this dataset represents a normalized lifecycle progress indicator based on cycle number, and was calculated as: DoD (%)* = (CycleNumber / 3000) × 100. This produces a value of approximately 0.03% for the first cycle and 100% for the final recorded cycle. It is important to note that this variable does not represent the physical Depth of Discharge of an individual charge-discharge cycle. It is a retrospective label that reflects the proportional position of each cycle within the 3000-cycle recorded lifecycle.

DoD(%)*=(CycleNumber/3000)×100

The column name DoD(%)* is retained for backward compatibility with the published Mendeley repository record. Users and researchers should treat this column as a normalized lifecycle progress indicator, not as a measurement of physical depth of discharge. It is perfectly collinear with CycleNumber and should not be used as an independent predictive feature in machine-learning experiments.

#### 4.1.7 Cell-voltage imbalance calculation

For visualization and descriptive analysis, the cell-voltage imbalance was calculated per cycle as the difference between the highest and lowest single cell voltages:

CellVoltageImbalance=Max(Cell1_VtoCell8_V)−−Min(Cell1_VtoCell8_V)

This derived variable was used only for analysis and visualization, and is not included in the published CSV dataset as an original column.

#### 4.1.8 Data preparation and organization

Following generation and curation, the battery-cycle records were organized into a structured tabular dataset. Each row is a battery cycle, and each column is a measured or calculated variable. The records were sorted sequentially by CycleNumber ranging from 1 to 3000. All the records were from the same pack so the battery pack was identified by PackID and kept the same. The variables carried by each record included Capacity (Ah), Voltage(V), Current(A), Temperature(C), and the eight individual cell-voltage variables, from Cell1_V to Cell8_V. The calculated variables SOH (%), RUL (cycles), and DoD (%) were added using the formulas given in 4.1.4, 4.1.5, 4.1.6. The dataset was reviewed to verify that all 3000 cycle records were present, that the cycle sequence was continuous, and that the necessary pack-level and cell-level variables were available. The final dataset was saved as comma-separated values with the file name of GMP_LFP_40Ah_3000Cycle.csv. Supporting documentation includes a codebook, README file and data publication permission letter [10].

SOH(%), RUL(cycles) and DoD(%)* are deterministic linear transformations of Capacity(Ah) and CycleNumber (SOH = Capacity/40 × 100; RUL = 3000 − CycleNumber; DoD = CycleNumber/3000 × 100), and Capacity(Ah) is itself a deterministic monotonic function of CycleNumber (Section 2.2.2). Any two of CycleNumber, RUL(cycles) and DoD(%) are therefore perfectly collinear (|r| = 1), as are Capacity(Ah) and SOH(%) (Fig. 4). Machine-learning researchers are advised: (i) not to use DoD(%), RUL(cycles) or CycleNumber as input features when the prediction target is RUL, SOH or Capacity, and not to use SOH(%) as a feature when Capacity(Ah) is the target, because this constitutes trivial target leakage and produces apparently perfect but meaningless accuracy; (ii) to define the prediction target from the capacity trajectory, for example the number of cycles until SOH falls below a threshold such as 80%, and to evaluate models with a chronological (cycle-ordered) train–test split rather than a random split; and (iii) to note that, because the operating variables were generated independently of the degradation trajectory (Table 5), they contain no information about capacity fade in this dataset, so the dataset is suited to the development, testing and teaching of data-handling, feature-engineering and modelling pipelines rather than to demonstrating that operating conditions predict degradation.

### 4.2 Data analysis methodology

Descriptive, correlation, outlier and graphical methods were applied to the data set. The analysis was performed on the 3000 cycle records in GMP_LFP_40Ah_3000Cycle.csv

#### 4.2.1 Data cleaning

The dataset was checked for missing values, duplicate rows, duplicate cycle numbers, and for non-numeric entries and discontinuities in the cycle sequence. There were no missing values and no duplicated rows. The CycleNumber values were sequential from 1 to 3000 for all 17 variables for each record.

The names, units and data types of the variables were also checked for consistency. The battery identifier was kept as PackID but was not used in the correlation and distribution analyses since it was identical for all records.

#### 4.2.2 Descriptive statistical analysis

For all numerical variables descriptive statistics were calculated. These were the minimum, maximum, mean and standard deviation. The statistics were used to summarize the ranges and variation of the degradation, pack-level, and cell-level variables that were recorded.

#### 4.2.3 Correlation analysis

Linear relationships were assessed by Pearson correlation coefficients between the numeric variables. The analysis was done using CycleNumber, Capacity (Ah), SOH (%), RUL (cycles), Voltage (V), Current (A), DoD (%), Temperature (C), Cell1_V to Cell8_V. In addition, Spearman rank correlation coefficients and mutual information (MI) scores were computed to assess monotonic and general (non-linear) dependence between the operating variables and Capacity(Ah)/CycleNumber (Table 5). MI was estimated with the k-nearest-neighbour estimator of Kraskov et al. as implemented in scikit-learn (mutual_info_regression, k = 3, random_state = 42) and is reported in nats.

The obtained coefficients were visualized in a correlation heatmap. PackID was excluded since a constant variable has no meaningful correlation coefficient.

#### 4.2.4 Outlier assessment

Individual cell voltage variables and the cell-voltage imbalance were evaluated for spread and possible outliers using boxplots (Fig. 5(a)) and were quantified with the Tukey rule, i.e. values below Q1 − 1.5·IQR or above Q3 + 1.5·IQR were counted per variable (Table 7). The flagged values were not removed: they lie well inside the specified individual cell voltage range (2.50–3.65 V) and, under the generation model of Section 2.2.2, they arise from the tails of the calibrated distributions rather than from data-entry or formatting errors.

#### 4.2.5 Cell-Voltage imbalance analysis

Cell-voltage imbalance for each cycle was calculated as the difference between the maximum and minimum voltage of Cell1_V to Cell8_V:

Cell voltage imbalance = Maximum cell voltage – Minimum cell voltage

The calculated imbalance was used only for descriptive analysis and visualization purposes. This was not added as an original column in the published CSV file.

#### 4.2.6 Lifecycle-phase statistics and normality testing

Descriptive statistics (mean, SD, skewness and IQR) were computed separately for the three lifecycle phases of Fig. 2 (Table 3) and individually for Cell1_V to Cell8_V (Table 4); cell uniformity was tested with one-way ANOVA, the Kruskal–Wallis test and Levene’s test. Normality of the distributions in Fig. 3 was tested with the Shapiro–Wilk test, the Kolmogorov–Smirnov test against a normal distribution with mean and SD estimated from the sample, and the D’Agostino–Pearson K² test, using SciPy (Table 6). Sample skewness and excess kurtosis are reported with bias correction. At n = 3000 these tests are sensitive to small departures from normality, so p-values should be read together with the skewness and kurtosis values.

**Fig. 2:**
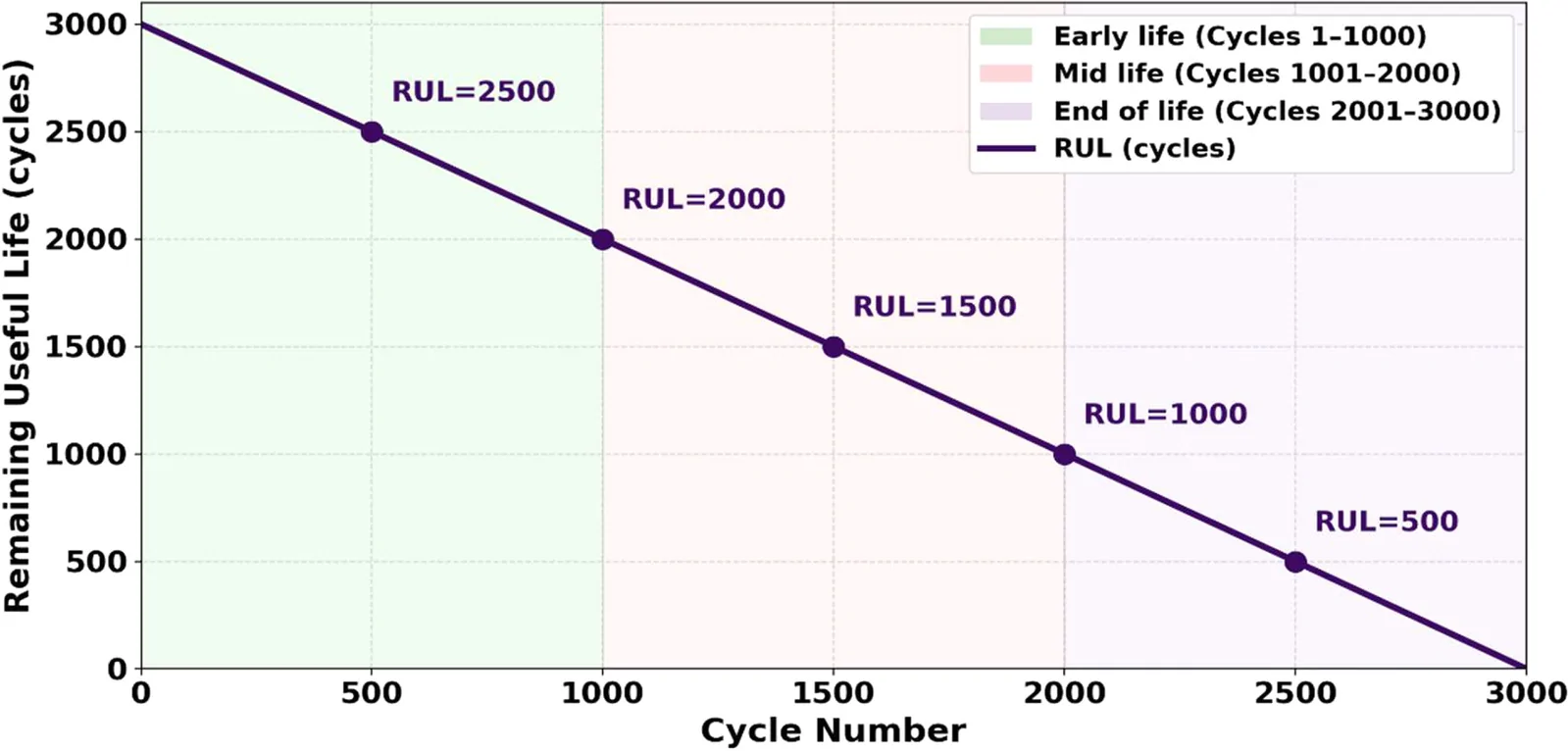
Remaining useful life progression with lifecycle Phase Zones. Note: RUL(cycles) is a retrospective label obtained by counting the cycles remaining to the last record in the sequence (RUL = 3000 − CycleNumber); it is not a probabilistic life-prediction result and carries no uncertainty (Section 4.1.5).

**Fig. 3:**
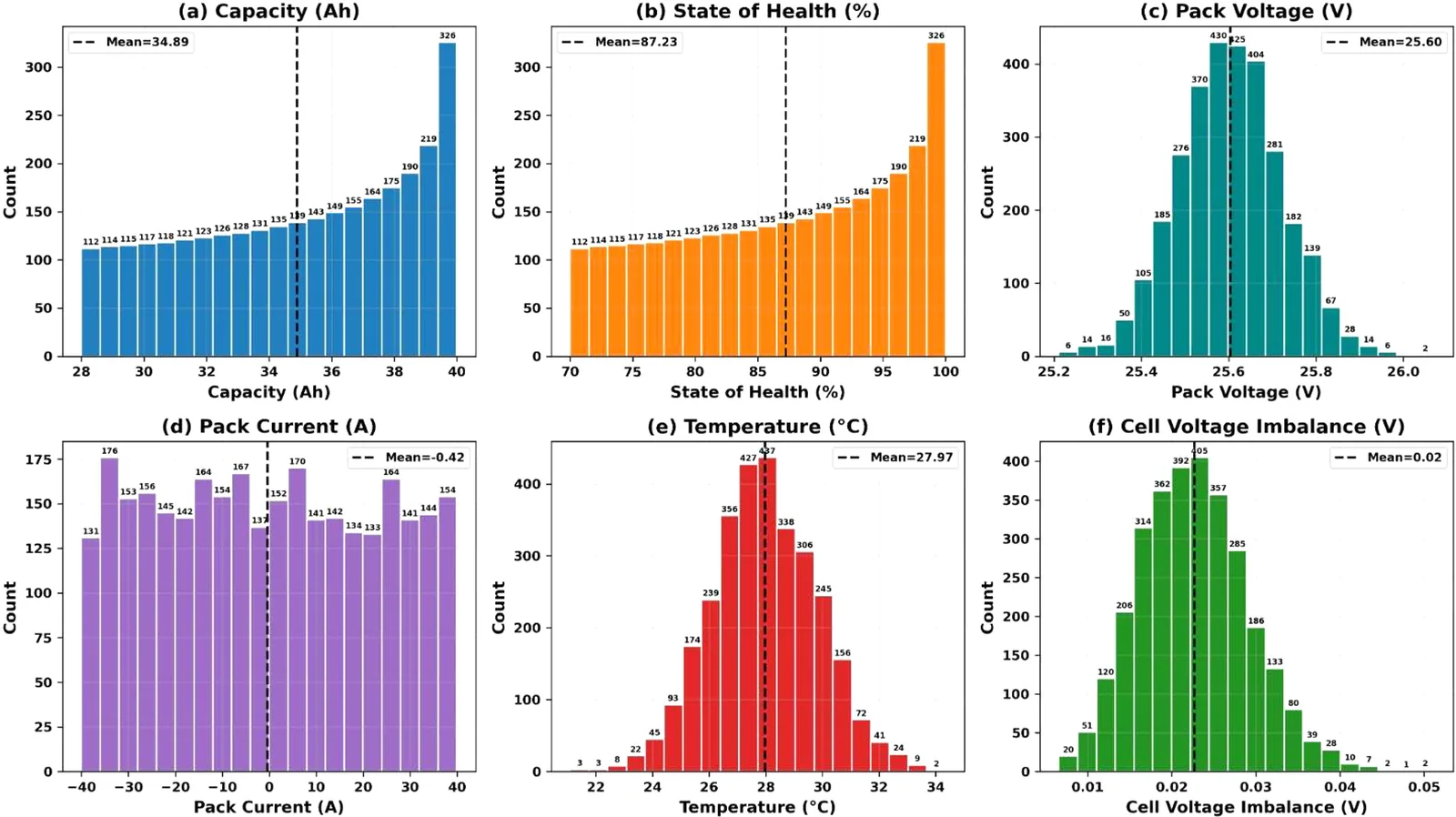
**(a)** Distribution of Capacity (Ah)**. (b**) Distribution of State of Health (%). **(c)** Distribution of Pack Voltage (V). **(d)** Distribution of Pack Current (A). **(e)** Distribution of Temperature ( °C). **(f)** Distribution of Cell-Voltage Imbalance (V) across 3000 recorded cycles.

### 4.3 Tools and software

The tools and software used for battery monitoring, data preparation, analysis and visualization are summarized in Table 8.

**Table 8:** Tools and software used for data acquisition and analysis.

Tool/Software	Description
LiFePO₄ battery pack	Industrial battery pack rated at 25.6 V and 40 Ah, consisting of eight series-connected cells.
Battery Management System	JK BMS manufactured by JIKONG, model JK_BD4A20S6P, configured for an 8S LiFePO₄ battery pack.
Active balancing system	Integrated active balancing with a rated balancing current of 0.4 A.
Current handling	BMS module label rating 60 A continuous and 100 A peak; configured pack limits 40 A continuous and 80 A peak per the GMP specification sheet. Label values, not independently verified.
BMS monitoring application	Manufacturer-supported application used for battery monitoring and access to BMS information.
Microsoft Excel	Used for initial inspection, tabular review, and checking of the dataset.
Python	Used for data preparation, statistical analysis, and visualization.
Pandas	Used to import, organize, inspect, and summarize the CSV dataset.
NumPy	Used for numerical calculations and derived-variable computation.
Matplotlib	Used to create line plots, histograms, scatter plots, boxplots, and correlation visualizations.
SciPy	Used for skewness/kurtosis, normality tests (Shapiro–Wilk, Kolmogorov–Smirnov, D’Agostino–Pearson), ANOVA, Kruskal–Wallis and Levene tests, and Spearman correlation.
scikit-learn	Used to estimate mutual information (mutual_info_regression, k-nearest-neighbour estimator).

### 4.4 Data quality control

Several checks were performed to verify the completeness and internal consistency of the dataset before publication. The dataset was inspected to confirm that all 3000 cycle records were present and that CycleNumber progressed continuously from 1 to 3000 without gaps or duplicate entries.

The dataset was also checked for missing values, non-numeric entries, duplicated rows, and inconsistent column names or units. All 17 variables were available for each recorded cycle. PackID remained constant across the dataset because all records belong to the same battery pack.

The calculated variables were verified using their defined relationships. SOH(%) was checked against Capacity(Ah) and the rated capacity of 40 Ah. RUL(cycles) was checked against the total lifecycle of 3000 cycles, and DoD(%) was checked against the normalized cycle progression used in the dataset.

The eight individual cell-voltage variables were reviewed for numerical consistency and comparable voltage ranges. Potential extreme values were retained because they lie inside the specified individual cell voltage range and arise from the calibrated distributions of Section 2.2.2 (Table 7).The final CSV file was compared with the codebook and README documentation to ensure that the variable names, units, descriptions, and file structure were consistent across the repository materials.

### 4.5 Data storage and sharing

The final dataset was saved in the comma-separated values format and named GMP_LFP_40Ah_3000Cycle.csv. Supporting documentation was created to enhance transparency and reusability. The files include a codebook of variables and units, a README file explaining the structure of the dataset and how to access it, and a permission letter to enable academic publication and public sharing of the industrial data, and the original manufacturer specification sheet of the battery pack.The dataset and supporting files were deposited in the Mendeley Data repository [10] with the title “Industrial LiFePO₄ Battery Management System Dataset for Early Degradation Analysis and Remaining Useful Life Prediction”. The dataset is available to public at following DOI: https://data.mendeley.com/datasets/k675pxrd83/2

The repository includes the following files:

1. GMP_LFP_40Ah_3000Cycle.csv -the dataset: 3000 cycle records × 17 variables, as described in Table 1(a).

**Table 1a:** Description of dataset variables.

Column name	Description	Unit	Data type	Example value
PackID	Identification number assigned to the battery pack	None	Integer	1
CycleNumber	Sequential number of the recorded battery cycle	Cycle	Integer	1
Capacity(Ah)	Available battery capacity associated with the cycle (deterministic degradation trajectory, Eq. (1))	Ah	Float	39.9998
SOH(%)	Battery State of Health relative to the rated capacity	%	Float	99.9994
RUL(cycles)	Retrospective countdown label: 3000 − CycleNumber. Represents cycles remaining to the last record in the sequence, not a probabilistic end-of-life estimate. Perfectly collinear with CycleNumber.	Cycles	Integer	2999
Voltage(V)	Pack-level operating voltage	V	Float	25.6596
Current(A)	Pack-level operating current. Positive and negative values indicate opposite current directions	A	Float	25.4992
DoD(%)	Normalized lifecycle progress indicator*, calculated as (CycleNumber / 3000) × 100. Values range from approximately 0.03% at Cycle 1 to 100% at Cycle 3000. NOTE: This variable represents normalized lifecycle progression and NOT the physical depth of discharge of an individual cycle. Column name DoD(%) is retained for repository compatibility. It is perfectly collinear with CycleNumber.	%	Float	0.0333
Temperature(C)	Operating temperature of the battery pack	°C	Float	27.6576
Cell1_V	Voltage of Cell 1	V	Float	3.2070
Cell2_V	Voltage of Cell 2	V	Float	3.2028
Cell3_V	Voltage of Cell 3	V	Float	3.2045
Cell4_V	Voltage of Cell 4	V	Float	3.2031
Cell5_V	Voltage of Cell 5	V	Float	3.2130
Cell6_V	Voltage of Cell 6	V	Float	3.2019
Cell7_V	Voltage of Cell 7	V	Float	3.1969
Cell8_V	Voltage of Cell 8	V	Float	3.1927
2. DataSet_CodeBook.pdf - codebook listing the variable names, units, data types and definitions, including the formulas of the calculated variables.
3. README.docx - general description of the dataset, its file structure, provenance and reuse instructions.
4. Data_Publication_Permission_Letter_GMP.pdf - Data_Publication_Permission_Letter_GMP.pdf written permission from GMP Lithium Ltd. authorising academic publication and public sharing of the dataset.
5. GMP_Specs_LFP_25.6V_40AH.pdf - GMP Industrial LFP Battery Original Manufacturer Specification Sheet for the GMP-LFP-2500 pack (25.6 V, 40 Ah, 8S LiFePO₄). Function: it documents the nominal, charge and operating voltage ranges, individual cell voltage limits, standard charge/discharge currents, BMS continuous and peak current limits, balancing current and operating temperature range, and is the source of the specification boundaries used to structure the dataset (Section 2.2.2).

The dataset is distributed under the Creative Commons Attribution 4.0 International licence.

## 5 Data Visualization

Several graphical representations were prepared to describe the structure and behavior of the dataset across the 3000 recorded cycles. These visualizations present the distribution, temporal progression, and relationships of the main degradation, operational, and cell-level variables.

Fig. 1 shows battery capacity (Ah) and State of Health (SOH, %) across 3000 recorded cycles on a dual-axis plot. Capacity declines from approximately 40 Ah to 28 Ah (left axis, blue) and SOH from 100% to 70% (right axis, orange, dashed), following a smooth, monotonic trajectory whose slope increases with cycle number (Eq. (1)).The commonly used 80% SOH threshold is marked with a horizontal reference line; it is crossed between Cycle 2221 and Cycle 2222.

Fig. 2 shows the Remaining Useful Life (RUL, cycles) decreasing linearly from 2999 at Cycle 1 to zero at Cycle 3000. The background is divided into three color-coded lifecycle phase zones early life (Cycles 1–1000), mid-life (Cycles 1001–2000), and end of life (Cycles 2001–3000) providing reference points for RUL prediction model evaluation and for the phase-wise statistics in Table 3. Key RUL values are annotated at every 500 cycles.

Fig. 3 presents a 2 × 3 histogram panel showing the frequency distribution of six key dataset variables across 3000 recorded cycles. Fig. 3**(a)** shows capacity (Ah) ranging from 28 Ah to 40 Ah with a mean of 34.89 Ah, reflecting the progressive capacity fade across the lifecycle. Fig. 3(**b)** shows State of Health (%) ranging from 70% to 100% with a mean of 87.23%, consistent with the capacity degradation trend. Fig. 3**(c)** shows pack voltage (V) approximately normally distributed around a mean of 25.60 V, within the expected operating range. current Fig. 3(d) shows pack current (A) distributed across both positive (charging) and negative (discharging) values with a mean of −0.42 A, reflecting the equal representation of charging and discharging records. Fig. 3(**e)** shows operating temperature ( °C) approximately normally distributed with a mean of 27.97 °C, with no trend across the lifecycle (Table 3). Fig. 3**(f)** shows cell-voltage imbalance (V) concentrated between 0.01 V and 0.05 V with a mean of 0.02 V, indicating a consistently small spread between the eight cells.A dashed vertical line marks the mean value in each panel, and each bar is labelled with its exact count. Formal normality tests for these six distributions are reported in Table 6.

Fig. 4 shows the Pearson correlation coefficient between the numerical variables in the dataset. PackID was excluded as it has the same value in all 3000 records. The variables in the heatmap are CycleNumber, the degradation-related variables, the pack-level operating variables, and the eight individual cell-voltage measurements. Correlation coefficients are measured on a scale of −1 to +1. A value close to +1 implies a strong positive linear relationship, a value close to −1 implies a strong negative linear relationship, and a value close to zero implies a weak linear relationship. Two high-correlation clusters are visible: CycleNumber, DoD(%)† and RUL(cycles) (|r| = 1.000), and Capacity(Ah) and SOH(%) (r = 1.000); the two clusters are themselves correlated at |r| = 0.995. All other coefficients satisfy |r| ≤ 0.05. Spearman and mutual-information analyses of the same relationships are given in Table 5.

**Fig. 4:**
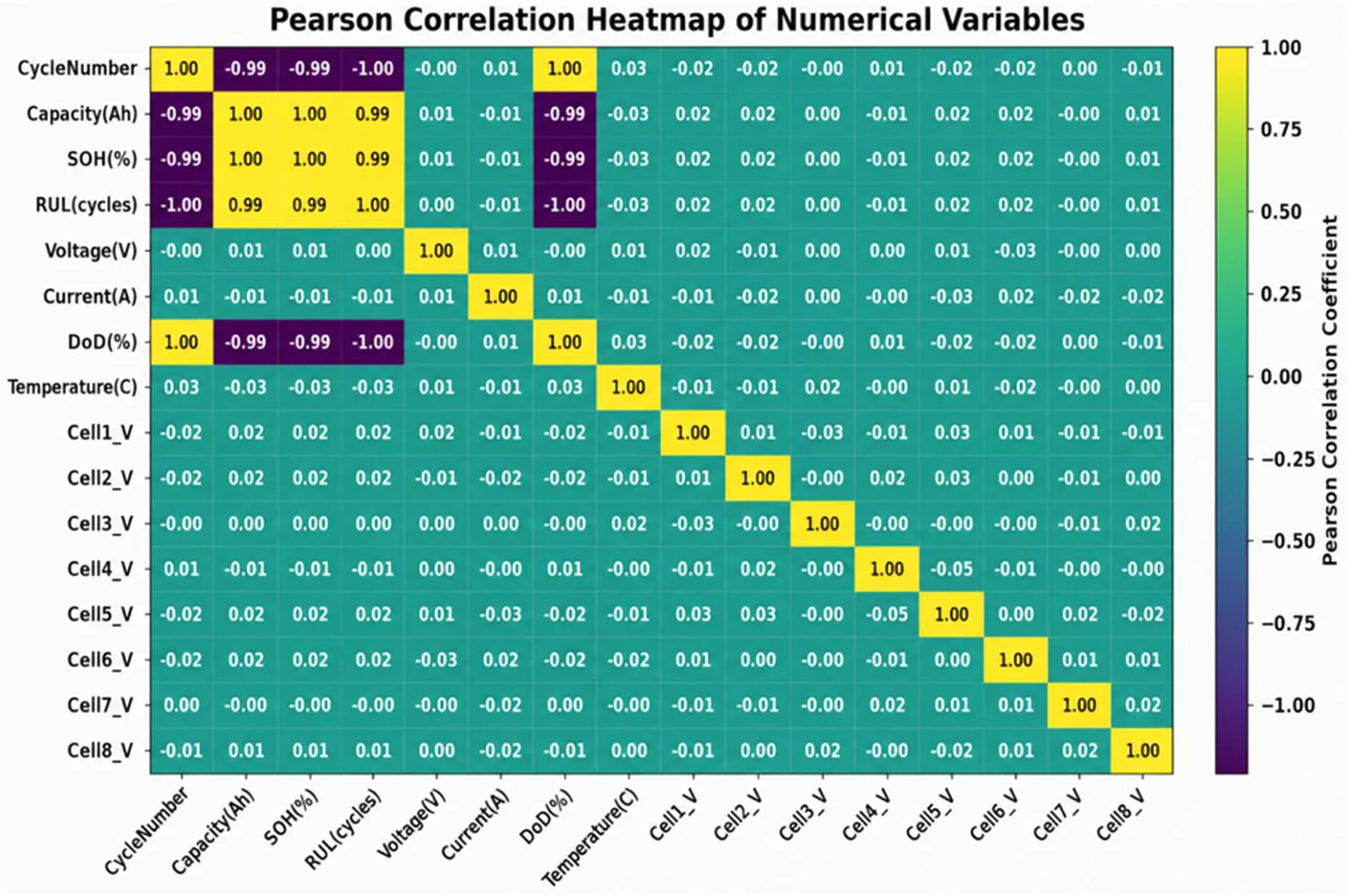
Pearson correlation heatmap of the numerical variables in the dataset. **High-correlation (collinear) clusters: (i) CycleNumber, DoD(%) and RUL(cycles)** (|r| = 1.000; exact linear transformations of one another); **(ii) Capacity(Ah) and SOH(%)** (r = 1.000; SOH = Capacity/40 × 100); and **cluster (i) with cluster (ii)** (|r| = 0.995; monotonic degradation trajectory). All remaining coefficients, involving Voltage(V), Current(A), Temperature(C) and Cell1_V–Cell8_V, satisfy |r| ≤ 0.05. The collinear variables in clusters (i) and (ii) must not be used together as predictors and target (Section 2.2.7).

Fig. 5 provides a two-panel cell-level voltage analysis. Fig. 5**(a)** shows color-coded boxplots for Cell 1 to Cell 8, with all cells exhibiting median voltages near 3.200 V and interquartile ranges between approximately 3.190 V and 3.210 V, indicating closely uniform voltage distributions across the eight cells (Table 4); the points beyond the whiskers are quantified in Table 7. Mean values (diamond markers) coincide across all eight cells Fig. 5(b) shows cell-voltage imbalance per cycle across the full 3000-cycle lifecycle, with data points colored by cycle number. The imbalance ranges from approximately 0.01 V to 0.05 V with a mean of 0.0227 V (dashed line), and the smoothed red trend line remains stable throughout the lifecycle, i.e. the imbalance shows no systematic change with cycle number (Table 3).

**Fig. 5:**
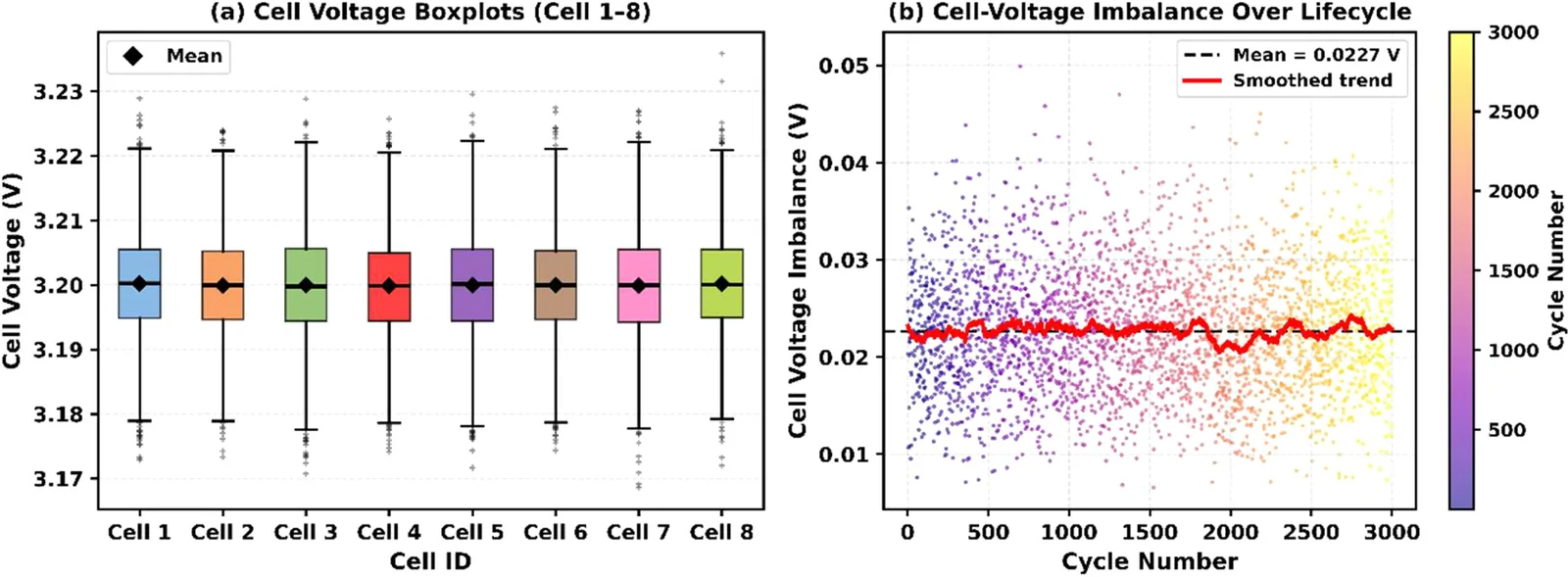
**(a)** Cell voltage boxplots for Cell 1–Cell 8 across 3000 recorded cycles. **(b)** Cell-voltage imbalance per cycle over the recorded lifecycle.

Fig. 6 presents two pack-level operating analyses. Fig. 6(a) against normalized lifecycle progress (DoD(%)*, colored by SOH, remaining within a narrow band of approximately 25.2 V to 26.1 V which corresponds to μV ± 3.5σV of Eq. (5). Fig. 6(b) shows operating temperature across 3000 cycles, ranging from approximately 21 °C to 34 °C, with no systematic change across cycles (Table 3).

**Fig. 6:**
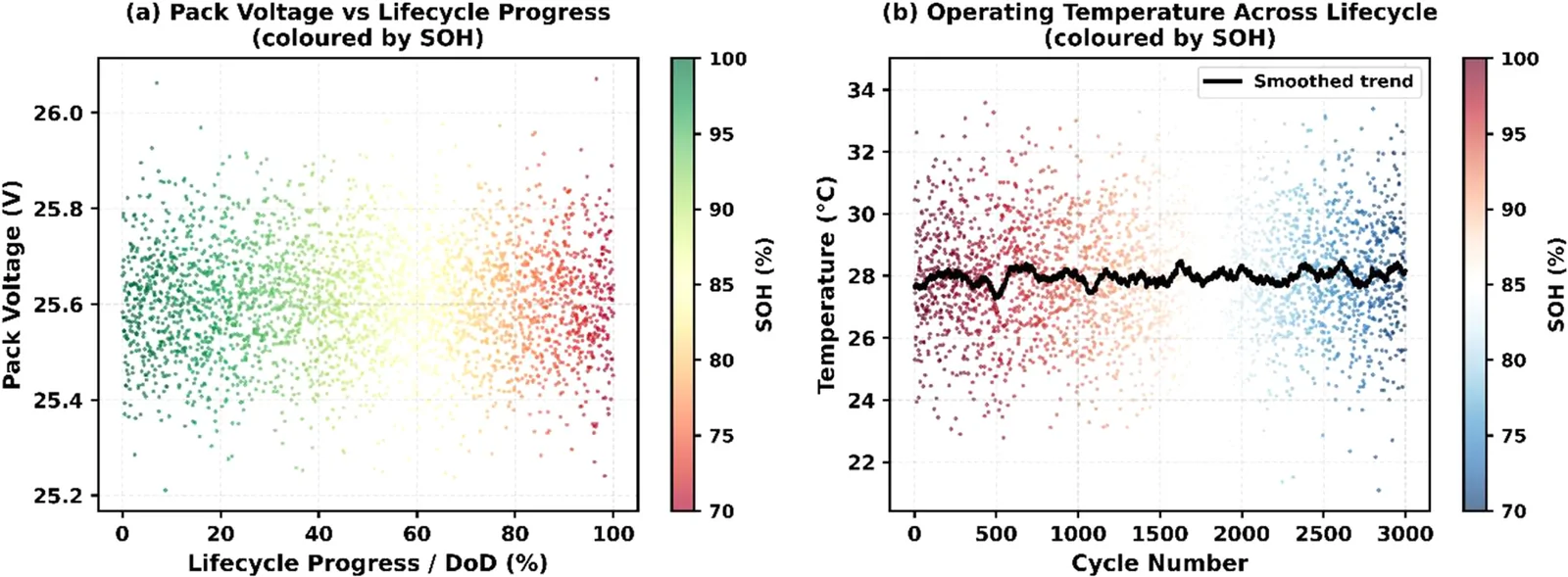
**(a)** Pack voltage versus normalized lifecycle progress (DoD%)* colored by SOH. **(b)** Operating temperature across 3000 cycles colored by SOH.

## Limitations

• The dataset is based on a single battery pack of one chemistry and configuration: LiFePO4, 25.6 V, 40 Ah, 8 cells in series. Models developed from this data alone may not generalize to other cell formats, nominal capacities, series or parallel configurations, battery manufacturers, or alternative chemistries such as NMC or NCA.
• The dataset contains a summary record at the cycle level for each of the 3000 cycles. High frequency intra-cycle voltage & current waveforms are not provided. So, the data does not support analyses that need detailed time behavior within a single charging or discharging cycle.
• The dataset is an experiment-informed R&D resource prepared by GMP Lithium Ltd. using knowledge from physical battery testing, BMS monitoring, charge-discharge equipment, accelerated ageing tests, and battery specifications. It is not a direct timestamp-by-timestamp export from a single continuous BMS logging session. Capacity(Ah) follows a deterministic degradation model, and the operating and cell-voltage columns are structured (simulated) values generated from the calibrated distributions of Section 2.2.2 independently of the degradation trajectory; they therefore contain no information about capacity fade (Table 5). Users should take this into account when applying the dataset to studies that require purely empirical or raw logged data.
• The records were prepared to reflect practical industrial operating conditions rather than a controlled laboratory ageing protocol. The DoD(%)* column represents a normalized lifecycle progress indicator based on cycle number and does not correspond to the physical depth of discharge of an individual charge-discharge cycle. Because the operating variables were generated independently of cycle number, the dataset cannot be used to isolate or quantify the contribution of current, temperature or cell imbalance to battery degradation.
• The RUL(cycles) label was assigned retrospectively according to the remaining number of cycles within the recorded 3000-cycle sequence. It is therefore a deterministic label based on the final available record and does not represent uncertainty in end-of-life detection or the probability distribution of the battery’s actual future service life. RUL(cycles) as defined here (3000 − CycleNumber) is perfectly collinear with CycleNumber. A machine-learning model trained to predict this RUL from CycleNumber alone would trivially solve the task. Researchers who wish to train meaningful RUL prediction models should use capacity-based or SOH-threshold-based end-of-life criteria (e.g., predicting the number of cycles until SOH falls below 80%) rather than treating the RUL column as an independent label.
• Cell-voltage columns (Cell1_V to Cell8_V) cells were generated from identical statistical distributions for all eight cells (Eq. (8)) and have identical means (3.200 V) and standard deviations (0.008 V) across the full lifecycle .They do not carry independent cell-level ageing or State-of-Charge signals. The dataset supports cell-voltage imbalance analysis (the spread across the eight cells per cycle) but should not be used to study individual cell degradation trajectories or cell-to-cell divergence over life.

## Ethics Statement

The authors have read and followed the ethical requirements for publication in Data in Brief. The current work does not involve human subjects, animal experiments, or data collected from social media platforms. The dataset consists of industrial battery operational and R&D records associated with a Battery Management System and does not contain personal, confidential, or identifiable information. GMP Lithium Ltd. has confirmed in writing that the dataset does not contain personal information, customer-identifiable information, confidential business information, commercial pricing information, or sensitive company information that would restrict its academic publication and distribution.

## Credit Author Statement

**MST. Asfia Binte Ulfat:** Conceptualization, Data Curation, Methodology, Formal Analysis, Software, Writing – Original Draft. **Afsana Begum:** Supervision, Validation, Review and Editing. **Md. Abdulla Al Mamun:** Supervision, Validation, Review. **Sawdagar Noor Asfuq E Azam:** Resources, Data curation (industrial battery specification and R&D data provision, GMP Lithium Ltd.). **Md. Mahedi Hassan:** Validation, Methodology, Review and Editing. **Imran Mahmud:** Supervision, Validation, Review. **A.H.M. Saifullah Sadi:** Supervision, Review and Editing. **Md. Shohel Sayeed:** Review and Editing.

## Appendix Supplementary Materials

Image, application 1
